# Percutaneous transforaminal endoscopic surgery (PTES) and mini-incision L5/S1 OLIF with a self-lock cage for the surgical treatment of L5 spondylolisthesis

**DOI:** 10.1186/s13018-023-04022-x

**Published:** 2023-07-24

**Authors:** Tianyao Zhou, Yutong Gu

**Affiliations:** 1 Department of Orthopaedic Surgery, Zhongshan Hospital Fudan University, Shanghai, China; 2 Shanghai Southwest Spine Surgery Center, Shanghai, China

**Keywords:** L5 spondylolisthesis, Minimally invasive surgery, Percutaneous transforaminal endoscopic surgery, Oblique lumbar interbody fusion, Mini-incision

## Abstract

**Objectives:**

We reported thirteen cases of percutaneous transforaminal endoscopic surgery (PTES) under local anesthesia and mini-incision L5/S1 OLIF (OLIF51) with a self-lock cage for the treatment of L5 spondylolisthesis.

**Methods:**

From Jan 2019 to Feb 2020, the patients with L5 spondylolisthesis with nerve root symptoms undergoing PTES and OLIF51 were included in this study. PTES under local anesthesia was performed in a prone position, and OLIF51 with a self-lock cage and allograft was then undertaken through a left abdominal mini-incision and oblique retroperitoneal approach between bilateral iliac vessels with the external oblique, internal oblique and transverse abdominal muscles bluntly separated in turn for L5/S1 in a right oblique position under general anesthesia. Back and leg pain were preoperatively and postoperatively evaluated using the VAS, and the clinical outcomes were evaluated with the ODI before surgery and at the 2-year follow-up. The anterior and posterior intervertebral space height (AISH, PISH), lumbar lordotic, and surgical segmental lordotic angle (SLA) were measured on lumbar spine X-rays preoperatively and postoperatively. The fusion status was assessed according to Bridwell’s fusion grades.

**Results:**

Thirteen cases of L5 spondylolisthesis were included. The operation duration was 49.1 ± 5.6 min for PTES and 73.6 ± 8.2 min for OLIF. There was blood loss of 25 (15–45) ml. The incision length was 7.5 ± 1.1 mm for PTES and 46.8 ± 3.8 mm for OLIF. The hospital stay was 5 (4–6) days, and the follow-up duration was 29 (24–37) months. For the clinical evaluation, the VAS of back and leg pain significantly dropped after surgery (*p *< 0.001), and the ODI significantly decreased from 64.7 ± 7.8% to 12.9 ± 4.3% 2 years after surgery (*p *< 0.001). AISH, PISH and SLA significantly improved after surgery (*p *< 0.05). Fusion grades based on the Bridwell grading system at the 2-year follow-up were grade I in 9 segments (69.2%) and grade II in 4 segments (30.8%). No patients had any form of permanent iatrogenic nerve damage or major complications. No failure of instruments was observed.

**Conclusions:**

PTES and mini-incision OLIF51 with a self-lock cage is a viable option of minimally invasive surgery for L5 spondylolisthesis, which can achieve direct neurologic decompression, satisfactory fusion and hardly destroys the rectus abdominis and its sheath, paraspinal muscles and bone structures.

## Introduction

Lumbar spine spondylolisthesis is a common disease and is mostly caused by lumbar degeneration or spondylolysis [1]. Lumbar interbody fusion is the most commonly used treatment for lumbar spondylolisthesis, including posterior lumbar interbody fusion (PLIF) [2, 3], transforaminal lumbar interbody fusion (TLIF) [4, 5], anterior lumbar interbody fusion (ALIF) [6, 7], and oblique lumbar interbody fusion (OLIF) [8–10]. For L5 spondylolisthesis, many studies of PLIF, TLIF and ALIF have been reported, but there are few studies of OLIF. Compared with L2-5 OLIF (OLIF25), the efficacy of indirect neurologic decompression of L5/S1 OLIF (OLIF51) through the approach between bilateral iliac vessels is less certain, and further posterior surgery is needed if neurologic symptoms do not improve. This leads to a longer operative time under general anesthesia and more invasiveness.

We used the hybrid surgery of percutaneous transforaminal endoscopic surgery (PTES) [11–13] combined with mini-incision L2-5 OLIF (OLIF25) and anterolateral screws rod fixation to treat L2-4 spondylolisthesis and achieved direct neurologic decompression and satisfying clinical outcomes with largely protected paraspinal muscles and bone structures [14, 15]. In this study, we reported thirteen cases of PTES under local anesthesia combined with mini-incision L5/S1 OLIF (OLIF51) using a self-lock cage for the treatment of L5 spondylolisthesis.

## Materials and methods

### Patients

This study was approved by the medical ethics committee of Zhongshan Hospital Fudan University, and the reference number is B2022-464R. Informed consent was obtained from all patients. From Jan 2019 to Feb 2020, the patients with L5 spondylolisthesis with nerve root symptoms undergoing PTES combined with OLIF51 using a self-lock cage through left lateral abdominal mini-incision and oblique retroperitoneal approach were included in this study.

**Fig. 1:**
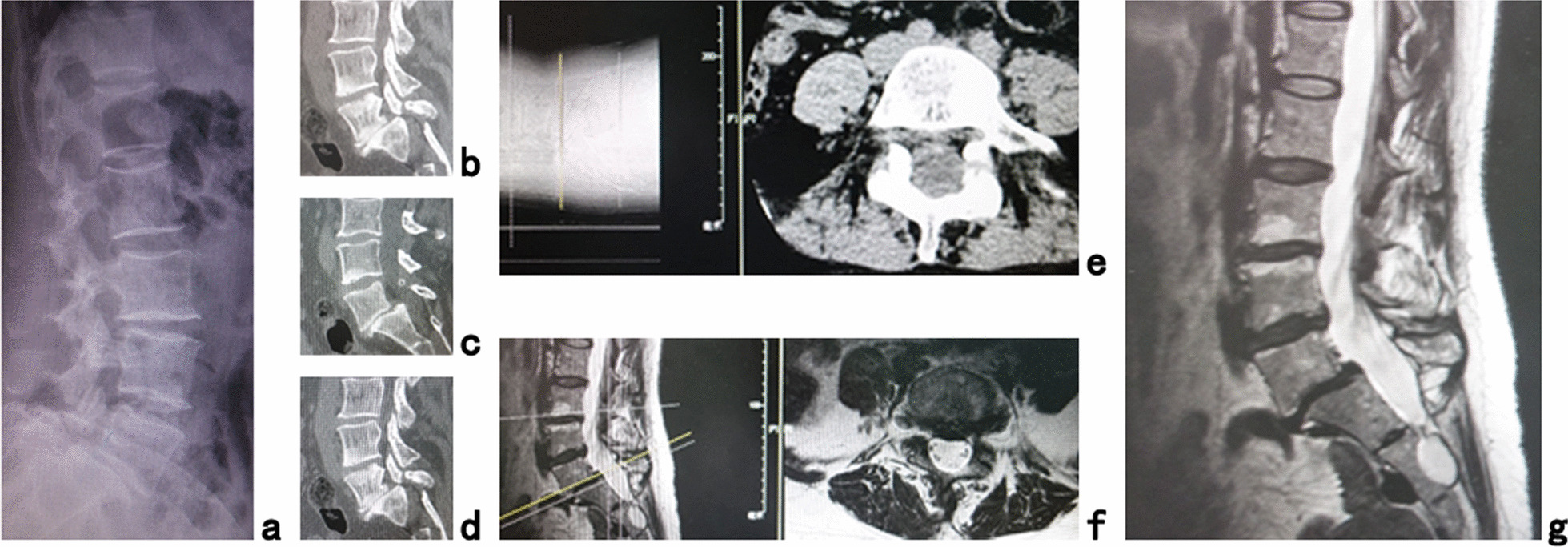
A 35-year-old male patient had low back pain and right leg pain for 1 year. **a** Lateral X-ray, **b**–**d** sagittal CT, **e** axial CT, **f** axial MRI and **g** sagittal MR images showed L5 spondylolisthesis (Meyerding II°) with bilateral spondylolysis and lateral recess stenosis at L5/S1 (Case 3)

The inclusion criteria were as follows: 1. Low back pain and unilateral or bilateral leg pain; 2. Image data of X-ray, MRI and CT showed L5 spondylolisthesis (Meyerding [16] I° or II°) corresponding to the neurologic findings (Fig. 1); 3. CTA showed the horizontal distance between the right-most border of the left common iliac vessel and the left-most border of the right common iliac vessel at L5/S1 > 25 mm; 4. Conservative treatment failed.

The exclusion criteria were the presence of more than 2-level lumbar spondylolisthesis, previous lumbar surgery, spinal infection or tumor, other medical conditions making the patient intolerant to operation, inability to provide informed consent, and a likelihood of noncompliance with follow-up.

### Pre- and postoperative imaging

All patients had a preoperative evaluation of CT and MRI imaging to determine L5 spondylolisthesis with disk herniation or lateral recess stenosis. Lateral X-rays were obtained to assess the slip degree of the vertebral body according to Meyerding [16]. Anterior and posterior intervertebral space height (AISH, PISH), lumbar lordosis (LL), and surgical segmental lordotic angle (SLA) were measured on lumbar spine X-rays preoperatively, postoperatively and at the 2-year follow-up. AISH and PISH: the perpendicular length from the anterior and posterior lower endplate of L5 to the upper endplate of S1 on the lateral X-ray; LL: the Cobb angle between the upper endplate of L1 and the upper endplate of S1; SLA: the Cobb angle between the superior endplate of L5 and S1 in the surgical segment. A loss of at least 2 mm of intervertebral space height is generally considered cage subsidence on X-ray[17]. The fusion status was assessed according to Bridwell’s fusion grades on CT [18].

### Surgical procedure

**Fig. 2:**
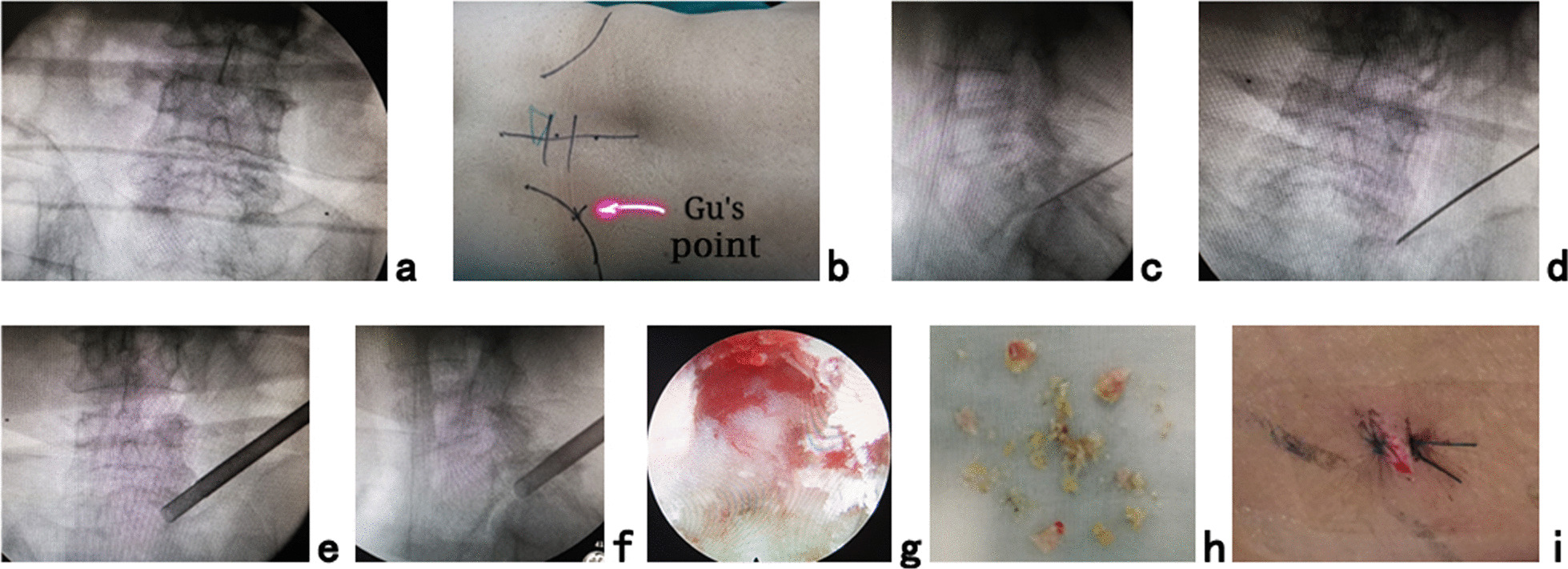
PTES for direct neurologic decompression in the surgical treatment of L5 spondylolisthesis. A transverse line bisecting the disk (L5/S1) was drawn along the metal rod, which was placed transversely across the center of the target disk on **a** the posteroanterior C-arm view in the prone position. **b** Photography showed the surface marking of the anatomic disk center identified by the intersection of the transverse line (L5/S1) and longitudinal midline, which was the aiming reference point of puncture, and the entrance point of puncture (Gu’s point) located at the corner of the flat back turning to the lateral side. During puncture, once resistance disappeared, the C-arm view was taken to ensure that the needle reached the target. The tip of the puncture needle was in the intracanal area close to the posterior wall of the disk on **c** lateral X-ray and near the lateral border of the pedicle on **d** posteroanterior X-ray. During press-down enlargement of foramen, when resistance disappears, the tip of the reamer should exceed the medial border of the pedicle on the **e** posteroanterior C-arm view and reach close to the posterior wall target disk on the **f** lateral C-arm view. Under **g** the endoscopic view, the compressed nerve root was freed after **h** the hypertrophic ligamentum flavum and herniated disk were removed. The right leg had a clear sense of relaxation. **i** The stab incision for PTES was approximately 8 mm (Case 3)

All surgeries were undertaken by the same senior surgeon (YT Gu). The C-arm was used for intraoperative fluoroscopic imaging. The patient was placed in a prone position with hyperkyphotic bolsters placed under the abdomen on a radiolucent table, especially in cases of the L5/S1 level with a high iliac crest. PTES was performed under local anesthesia with conscious sedation. The entrance point of the puncture was located at the corner of the flat back turning to the lateral side at the height of the target disk or cranially or slightly caudally, which is named “Gu’s point” [11–13]. An 18-gauge puncture needle was inserted anteromedially at an angle of approximately 45° (25°-85°) to the horizontal plane, aiming at the vertical line through the intersection of the posterior midline and target disk transverse line (Fig. 2a, b). After successful puncture (Fig. 2c, d) and stepwise dilation, press-down enlargement of foramen was performed using a 7.5-mm diameter hand reamer through an 8.8-mm diameter cannula docked at the facet joint [11–13]. When resistance disappears, the tip of the reamer should exceed the medial border of the pedicle on the posteroanterior view and reach close to the posterior wall of the target disk on the lateral view. (Fig. 2e, f) Through a 7.5-mm diameter working cannula, the compressed nerve root, even the contralateral nerve root, was freed (Fig. 2g) after the herniated disk and hypertrophic ligamentum flavum (Fig. 2h) were removed under endoscopy. The involved legs had an apparent sense of relaxation after neurologic decompression was achieved. The stab incision for PTES was approximately 8 mm (Fig. 2i).

**Fig. 3:**
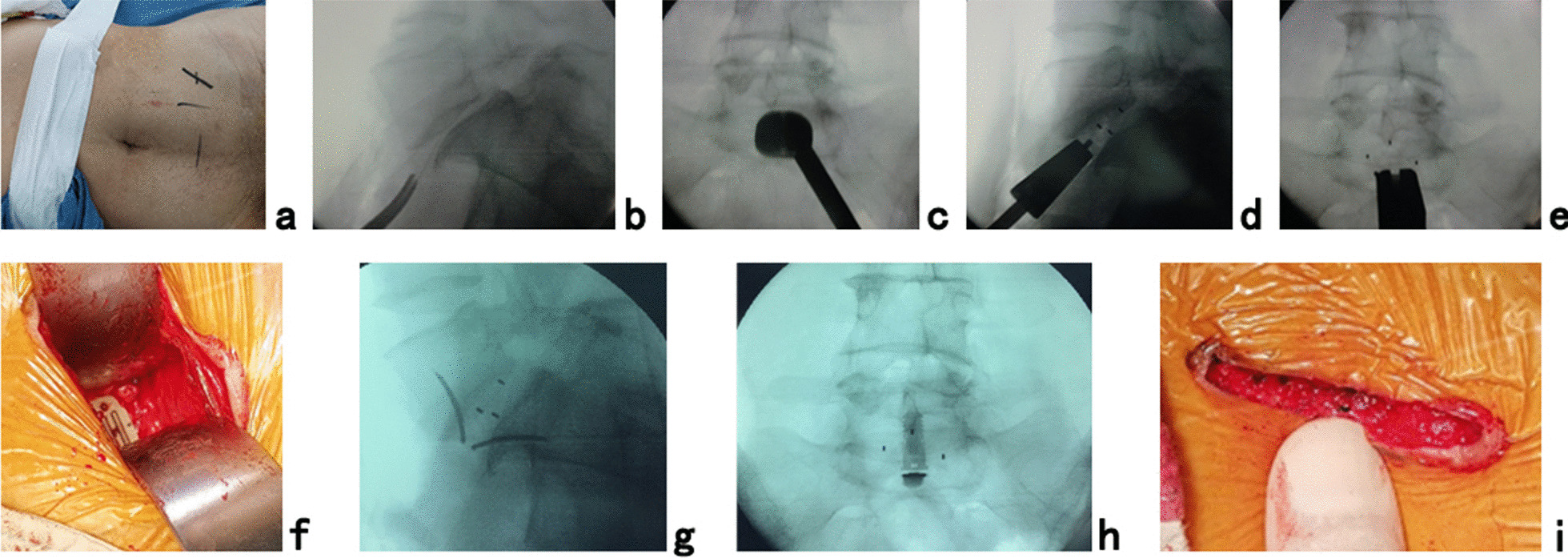
OLIF with a self-lock cage through a mini-incision for the surgical treatment of L5 spondylolisthesis. **a** The patient was placed into a right oblique position. The L5/S1 intervertebral space was positioned using **b** C-arm view. **c** X-ray view showing trial molding after discectomy. **d** Lateral and **e** posteroanterior C-arm views were used to check the position of the self-lock cage placed into the disk space parallel to the endplate. **f** The picture shows the surgical field after two self-lock anchors were inserted from the cage into the vertebrae. **g** Lateral and **h** posteroanterior C-arm views confirmed good positioning of the internal instruments. **i** The photograph shows the mini-incision for OLIF (Case 3)

Then, the patients were placed into a right oblique position under controlled general anesthesia with a tracheal cannula to undergo OLIF51 with a self-lock cage. The mini-incision was located inferomedially along the midline between the lateral edge of the rectus and the anterior superior spine of the iliac crest at the level of the midline between the umbilicus and symphysis pubis in the left lateral abdomen (Fig. 3a). After the skin and subcutaneous tissues were incised, the external oblique, internal oblique and transverse abdominal muscles were bluntly separated in turn to enter the retroperitoneal space and expose the anterior border of the left psoas and bilateral iliac vessels with two narrow long retractors. After fluoroscopic projection to confirm the L5/S1 segment (Fig. 3b), the intervertebral fibrous annulus was opened anteriorly between the bilateral iliac vessels. The intervertebral tissue was removed, and the upper and lower cartilage endplates were adequately scraped off, taking care to avoid damaging the bony endplates during the operation. After trial molding (Fig. 3c), a self-lock cage (Roi-A, Zimmer, USA) of appropriate size was filled with allograft bone and autograft obtained during PTES and obliquely placed into the disk space parallel to the endplate. Then, the holder of the cage was rotated right until parallel to the sagittal plane, and a fluoroscopic view was taken to confirm the good position of the cage (Fig. 3d, e) before two self-lock anchors were inserted from the cage into the vertebrae (Fig. 3f–h). Finally, the surgical incision (Fig. 3i) was closed layer by layer with a thin drain tube.

Patients could walk with a flexible brace after the drain tube was removed, usually 1 or 2 days after surgery, when the drainage fluid was less than 20 ml/24 h. After leaving the hospital, patients were encouraged to return to daily life and were followed up regularly.

### Clinical follow-up

The operation duration, frequency of intraoperative fluoroscopy, blood loss, incision length and hospital stay were recorded. Back and leg pain were evaluated using the 10-point visual analog scale (VAS) preoperatively; immediately; 1, 2, 3 and 6 months; and 1 and 2 years after surgery. The clinical outcomes were evaluated with the Oswestry Disability Index (ODI) preoperatively and at the 2-year follow-up. Each of the 10 questions is scored from 0 to 5, giving a maximum score of 50. The total score is then converted into a percentage of 50, which is ODI. During the follow-up, all complications were recorded, including iatrogenic nerve damage, vascular injuries, infection, wound healing, thrombosis, or recurrence.

### Statistical analysis

All data were analyzed by SPSS version 20.0 software, and a value of less than 0.05 was considered statistically significant. Normal distributed continuous variables such as age, operative duration, blood loss, incision length, follow-up and ODI were presented as mean ± standard deviation (SD); Discrete, rating variables and continuous variables, which are not normally distributed, were presented as median (Maximum- Minimum) including fluoroscopy frequency, hospital stay, VAS; Categorical variables such as gender were expressed as frequency or percentage. One-way ANOVA was used for AISH, PISH, LL and SLA comparisons at different time points. Comparison of VAS at different time points was performed using Kruskal–Wallis test followed by the Dunn procedure with Bonferroni correction. The ODI scores before treatment and 2 years after surgery were compared by matched samples *t* tests.

## Results

Thirteen cases of L5 spondylolisthesis with nerve root symptoms were included in the present study. There were 8 women (8/13, 61.54%) and 5 men (5/13, 38.46%) with a mean age of 53.38 ± 12.89 years. All patients were successfully treated using the surgical method described. The patients’ characteristics are summarized in Table 1.

**Table 1:** Summary of the clinical data of the patients

Case No.	Age (years), sex	Meyerding grade	Cause of disease	Fusion grade
1	71, F	II	Spondylolysis	I
2	52, F	I	Degeneration	I
3	35, M	II	Spondylolysis	I
4	60, M	II	Spondylolysis	II
5	57, M	I	Spondylolysis	I
6	39, F	I	Spondylolysis	I
7	68, F	II	Spondylolysis	I
8	74, M	I	Spondylolysis	II
9	57, F	II	Spondylolysis	I
10	37, M	I	Spondylolysis	I
11	45, F	II	Spondylolysis	II
12	44, F	I	Spondylolysis	I
13	55, F	II	Spondylolysis	II

The operation duration was 49.08 ± 5.57 min for PTES and 73.62 ± 8.24 min for OLIF. The frequency of intraoperative fluoroscopy was 5 (5–7) times for PTES and 5 (4–8) times for OLIF. There was blood loss of 27.69 ± 8.57 ml. The incision length was 7.46 ± 1.13 mm for PTES and 46.85 ± 3.80 mm for OLIF. The hospital stay was 5 (4–6) days (Table 2).

**Table 2:** Perioperative data

	PTES	OLIF51
Operation duration (min)	49.08 ± 5.57	73.62 ± 8.24
Frequency of intraoperative fluoroscopy (times)	5 (5–7)	5 (4–8)
Incision length (mm)	7.46 ± 1.13	46.85 ± 3.80
Blood loss (ml)	27.69 ± 8.57	
Hospital stay (days)	5 (4–6)	

The follow-up duration was 29.85 ± 4.06 months. The VAS of the back dropped significantly from 7 (6–10) preoperatively to 2 (0–3) immediately after surgery and to 0 (0–1) at the 2-year follow-up (*P *< 0.001). The VAS of the leg dropped from 8 (7–10) preoperatively to 1 (0–3) immediately after surgery and to 0 (0–2) at the 2-year follow-up (*P *< 0.001) (Table 3). The ODI significantly decreased from 64.68 ± 7.78 to 12.91 ± 4.31 2 years after surgery (*P *< 0.001) (Table 3).

**Table 3:** Variations in VAS, ODI(%) and radiological parameters

	Pre-op	Post-op	1 month	2 months	3 months	6 months	1 year	2 years
Back pain VAS	7 (6–10)	2 (0–3)^#^	0 (0–2)^#^	0 (0–2)^#^	0 (0–1)^#^	0 (0–1)^#^	0 (0–1)^#^	0 (0–1)^#^
Leg pain VAS	8 (7–10)	1 (0–3)^#^	1 (0–2)^#^	0 (0–2)^#^	0 (0–2)^#^	0 (0–2)^#^	0 (0–2)^#^	0 (0–2)^#^

	Before operation	2 years after operation	*P* value
ODI	64.68 ± 7.78	12.91 ± 4.31^#^	< 0.001

	Before operation	Immediately after operation	2 years after operation	*P* value
AISH (mm)	9.72 ± 4.18	16.05 ± 0.99^#^	15.49 ± 1.02^#^	< 0.001
PISH (mm)	2.82 ± 1.39	5.52 ± 2.08^#^	5.05 ± 2.02^#^	< 0.001
LL (°)	40.40 ± 7.46	44.16 ± 7.59	43.62 ± 7.53	0.397
SLA (°)	12.90 ± 7.22	19.77 ± 6.79*	17.98 ± 5.37*	0.031

^#^*P* < 0.001, significant difference between preoperatively and postoperatively

*P < 0.05, significant difference between preoperatively and postoperatively

**Fig. 4:**
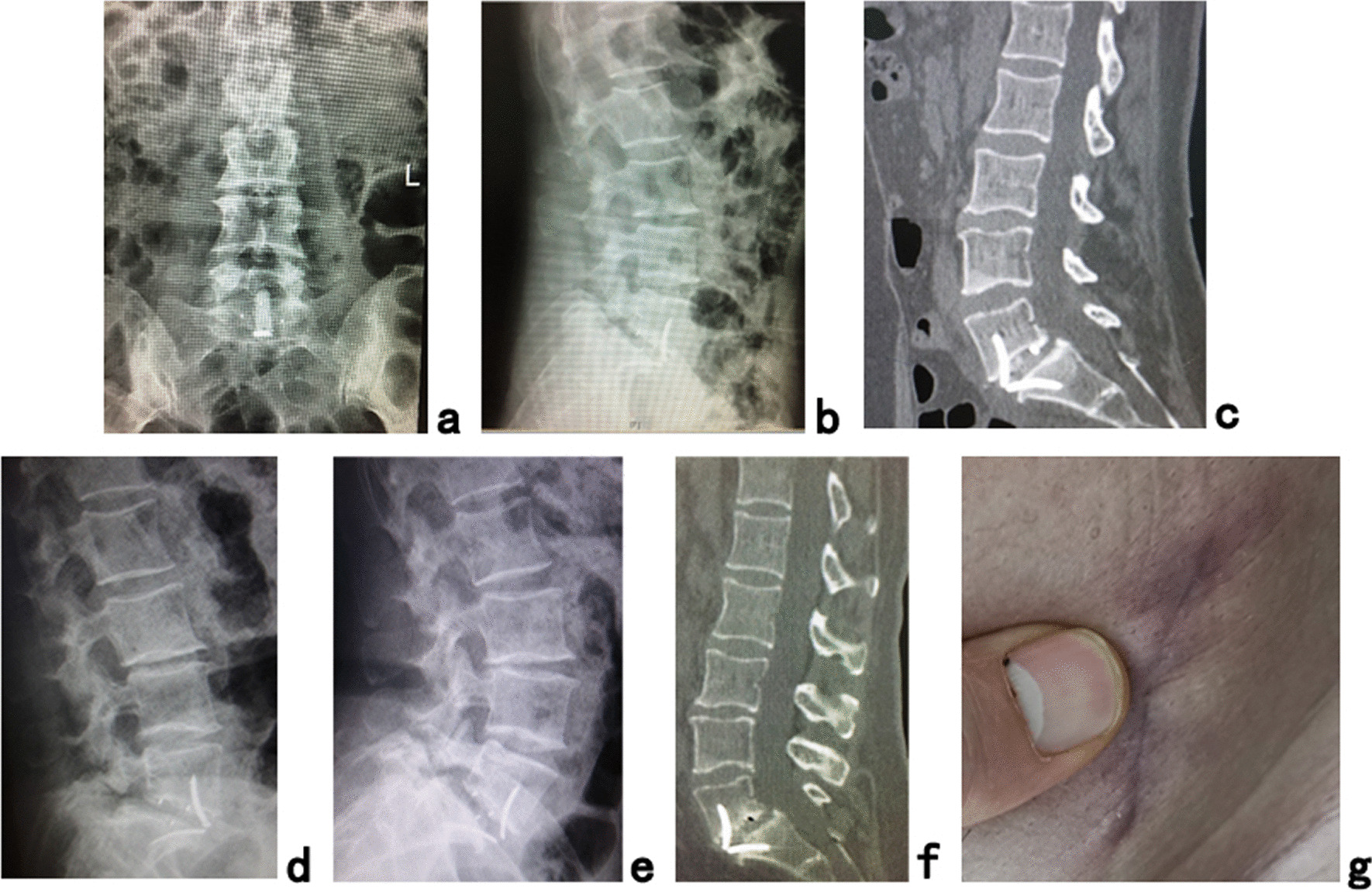
**a** Posteroanterior and **b** lateral X-ray images and **c** sagittal CT image showing good positioning of the cage immediately after the operation. No instability at the fusion level was found on **d** hyperflexion and **e** hyperextension lateral X-ray images. The fusion grade at the 2-year follow-up was grade I on the **f** sagittal CT image. **g** The picture shows the cosmetic incisions for OLIF51 (Case 3)

The postoperative radiographs and CT scans demonstrated good positioning of the cage (Fig. 4a–c). Postoperative AISH, PISH and SLA were 16.05 ± 0.99 mm (*P *< 0.001), 5.52 ± 2.08 mm (*P *< 0.001) and 19.77 ± 6.79° (*P *< 0.05), respectively, which were significantly greater than those preoperatively. Preoperative and postoperative LL were 40.40 ± 7.46° and 44.16 ± 7.59°, respectively, and there was no significant difference. No significant changes in AISH, PISH, LL, SLA and no instability at the fusion level were observed 2 years after surgery (Fig. 4d, e). Fusion grades based on the Bridwell grading system at the 2-year follow-up were grade I (Fig. 4f) in 9 segments (69.2%) and grade II in 4 segments (30.8%) (Table 1). No subsidence of cages and no failure of instruments were observed. There were no patients with any form of permanent iatrogenic nerve damage or major complications.

## Discussion

For the treatment of L5 spondylolisthesis, ALIF had some advantages, such as no damage to paraspinal muscles and bone structures, less blood loss, faster recovery, a larger cage with a possible higher fusion rate because of the greater contact surface between the endplate of the vertebra and cage and more graft bone as compared with PLIF or TLIF [10, 19–21]. Many studies have proven that ALIF can achieve similar or better clinical outcomes than PLIF or TLIF [20, 22, 23]. ALIF was performed through the abdominal paramedian retroperitoneal approach in the supine position, which may damage the rectus abdominis or its sheath. In this study, we undertook OLIF51 between the bilateral iliac vessels through an abdominal oblique retroperitoneal approach in a right oblique position, and the mini-incision was located in the left lateral abdomen with the external oblique, internal oblique and transverse abdominal muscles bluntly separated in turn for L5/S1. This method can protect the rectus abdominis and its sheath and paraspinal muscles, and the right oblique position allows the abdominal contents to fall away from the operative field and the left iliac vessels and psoas to be exposed clearly, which can guarantee operative safety. The results showed that there was no injury to the blood vessels, ureter or abdominal organs. Different from OLIF through the corridor between the psoas and the great vessels in the segments above L5, particular attention should be given to OLIF51 because the level of bifurcation of the great vessels also affects access at L5/S1 [8]. Approximately 28.3% of the population is not suitable for OLIF through the corridor between bilateral iliac vessels at L5/S1 because the entrance is obstructed by the great vessels [24]. Therefore, preoperative CTA is critical to assess the feasibility of the operation.

The self-lock cage of ALIF was used in OLIF51 in this study and can provide immediate stability and restoration of the lumbar anatomy sequence. AISH, PISH, and SLA significantly improved after surgery (*p *< 0.001). Fusion was achieved in all patients at the 2-year follow-up, and there was no failure of instruments. No subsidence of the cage into the vertebral body was found, which was related to protection of the cortical endplate during preparation of the intervertebral space. The results of the study showed that AISH and PISH significantly increased from 9.72 ± 4.18 mm and 2.82 ± 1.39 mm, respectively, to 16.05 ± 0.99 mm (*P *< 0.001) and 5.52 ± 2.08 mm (*P *< 0.001) after surgery. However, the difference in posterior height of the L5/S1 space between preoperative and postoperative evaluations was much less than that of anterior height, which was related to the cage inserted into L5/S1 from the front to back and the posterior disk space not being sufficiently distracted. This unbalanced distraction of L5/S1 could not tighten the posterior longitudinal ligament, enlarge the cross-sectional area (CSA) of the spinal canal and intervertebral foramen or alleviate the pressure on neurologic elements, which made the efficacy of indirect decompression in OLIF51 unsatisfying. Due to the large cage placed into the disk space from the lateral side to distract the anterior and posterior disk space balancedly in OLIF25 [25–30], the indirect decompression of OLIF25 is better than that of OLIF51.

If neurologic symptoms were not improved after OLIF51, further posterior surgery was needed for direct decompression, which sharply reduced the advantages of OLIF because of the longer operative time under general anesthesia and more damage. We performed PTES [11–13] under local anesthesia combined with OLIF51 for the treatment of L5 spondylolisthesis. PTES is a transforaminal endoscopic surgical technique with reduced steps, simple orientation and easy puncture, which can significantly decrease the times of fluoroscopy projection and shorten the operation duration [11–13]. The puncture point of PTES is located at the corner of the flat back turning to the lateral side, named “Gu’s Point” [11–13], which does not depend on X-ray fluoroscopy, distance measurement, age, gender or body size. Gu’s point is more medial than other transforaminal endoscopic techniques and has four advantages: (1) it avoids injuring the exiting nerve root; (2) it avoids blockage by the high iliac crest for the L5/S1 level; (3) it shortens the manipulation path, especially in obese patients; and (4) it avoids injuring abdominal viscera and great vessels [11–13]. During the PTES procedure, we performed press-down enlargement of foramen to remove the ventral bone of the articular process so that the working channel could be inserted into the spinal canal even if the puncture angle was 85° to the horizontal plane [11–13]. In addition, the hypertrophic ligamentum flavum and the protruding nucleus pulposus were removed to expand the lateral recess and decompress the ipsilateral traversing and exiting nerve root. The contralateral traversing nerve roots can also be exposed to enlarge the central spinal canal, and the bilateral nerve roots can be decompressed through a unilateral approach in a small incision. PTES before OLIF can achieve direct decompression and avoid another entrance into the operation room. Reoperation, even PTES, might put more psychological pressure on patients and surgeons, especially in China, where the doctor‒patient relationship is sometimes challenging. The results of this study showed that the VAS score of leg pain significantly dropped after surgery, the ODI score was significantly reduced 2 years after surgery, and there was no reoperation for neurologic decompression.

The PTES technique under local anesthesia was used to achieve direct decompression with minimal trauma, little blood loss and no more operative time of general anesthesia added to OLIF. Compared with general anesthesia, local anesthesia had little influence on physical status. This combination of two minimally invasive surgeries protected the paraspinal muscles and bone structures as much as possible, and there was only little blood loss and two small incisions (Fig. 4g). The frequency of intraoperative fluoroscopy during the operation was limited, and both the patients and surgeons were protected against radiation exposure. The natural corridor for OLIF51 and the self-lock cage led to minimal postoperative drainage fluid; when it was less than 20 ml/24 h, the drain tube was removed usually 1 or 2 days after surgery and patients could leave the hospital as soon as possible. In this study, no patients had any form of permanent iatrogenic nerve damage or major complications. All these confirmed the safety of the combination of two minimally invasive surgeries.

## Conclusions

PTES and mini-incision OLIF51 with a self-lock cage is a viable option of minimally invasive surgery for L5 spondylolisthesis, which can obtain direct neurologic decompression, satisfying fusion, and largely protect the rectus abdominis and its sheath, paraspinal muscles and bone structures.

## Data Availability

The data that support the findings of this study are available from the corresponding author, Yutong Gu, upon reasonable request.
